# Intra- and extra-familial adverse childhood experiences and a history of childhood psychosomatic disorders among Japanese university students

**DOI:** 10.1186/1751-0759-1-9

**Published:** 2007-04-02

**Authors:** Akinori Masuda, Takao Yamanaka, Tadatoshi Hirakawa, Yasuyuki Koga, Ryosuke Minomo, Takao Munemoto, Chuwa Tei

**Affiliations:** 1 Masuda Clinic, 1-30 Yamanoguchi, Kagoshima 892-0844, Japan; 2 National Institute of Fitness and Sports in Kanoya, 1 Hakusui-cho, Kanoya 891-2393, Japan; 3 Social and Behavioral Medicine, Kagoshima University, 8-35-1 Sakuragaoka, Kagoshima 890-8520, Japan; 4 Nishi-kyushu University, 4490-9 Ozaki, Kanzaki-cho, Saga 842-8585, Japan; 5 Kagoshima International University, 8850 Shimofukumoto-cho, Kagoshima 891-0191, Japan; 6 Cardiovascular, Respiratory and Metabolic Medicine, Kagoshima University, 8-35-1 Sakuragaoka, Kagoshima 890-8520, Japan

## Abstract

**Background:**

Japan has been witnessing a considerable increase in the number of children with psychosomatic disorders. The purpose of this study is to examine the relationship between the risk of psychosomatic disorder in adolescents and intra- and extra-familial adverse childhood experiences (ACEs).

**Methods:**

A retrospective cohort study of 1592 Japanese university students (52% male, mean age 19.9 years) who completed a survey about intra- and extra-familial ACEs and the incidence of childhood psychosomatic disorders. Intra-familial ACEs included domestic violence, physical violence, emotional abuse, illness in household, parental divorce, no parental affection, and dysfunctional family. Extra-familial ACEs included physical violence or negative recognition by teachers, being bullied in elementary or junior high school, or sexual violence.

**Results:**

The frequency of psychosomatic disorders among the respondents was 14.8%. Among the 7 intra-familial ACEs, emotional abuse (relative risk, RR = 1.9) and illness in household (RR = 1.7) increased the risk of psychosomatic disorders. Estimates of the relative risk for the 5 extra-familial ACEs were statistically significant and ranged from 1.5 for being bullied in elementary school or physical violence from teachers to 2.4. Students who had 3 or more intra-familial ACEs and 2 or more extra-familial ACEs had a 3.0 relative risk for psychosomatic disorder.

**Conclusion:**

These results suggest that intra- and extra-familial ACEs are associated with the development of psychosomatic disorders. Therefore, sufficient evaluation of ACEs should be performed in adolescent patients with psychosomatic disorder.

## Background

From the 1990s, Japan has been witnessing a considerable rise in the number of children with psychosomatic disorders [1]. Among children who consulted medical institutions in 1999, the number of children with psychosomatic disorders such as irritable bowel syndrome, headache, hyperventilation syndrome, bronchial asthma, eating disorder, and orthostatic dysregulation etc., increased with age and peaked at the age of 14 in males (18.3%) and at 15 in females (26.8%) [2] due to stress arising from academic competition and interpersonal relations with classmates or teachers in school [3] or due to poor parental interaction [4].

In the USA, it was reported that poverty and family problems produced by social structure are important causes of school childrens' health-care problems [5]. Our recent study demonstrated that 20–30% of adolescent patients who consulted psychosomatic clinics in Japan had various family problems that included being physically or mentally abused by family members, parental separation or divorce, and/or family dysfunction [6].

Many researchers have reported that childhood trauma and adverse experiences lead to a variety of negative health outcomes, including depressive disorder, attempted suicide, eating disorder, alcohol abuse, and anxiety disorder among adolescents and adults [7-10]. However, there is little information about the relationship between multiple childhood adverse experiences and the risk of psychosomatic disorder in Japanese adolescence.

Psychosomatic symptoms are frequently reinforced when adolescents experience failure in school and social and emotional conflict in their relationships with parents [11]. In this study, we classified adverse childhood experiences (ACEs) into intra- and extra-familial ACEs and then evaluated these ACEs as risk factors for the development of psychosomatic disorders.

## Methods

### Subjects

A questionnaire survey on occurrence and risk for the development of psychosomatic disorder was carried out among 1636 first- and second-year Japanese university students from April to May 2002. Original questionnaire data were obtained from 1592 students (valid response rate: 97.3%). Of these, 831 (52.2%) were males and 761 (47.8%) were females. The mean age was 19.9 years (standard deviation = 1.9 years), range 18 – 23 years. After obtaining informed consent, the self-reporting questionnaire survey was conducted before interview-based confirmation of the details.

### Procedures

#### Definition of psychosomatic disorder

Diseases such as non-ulcer dyspepsia, peptic ulcer, headache including migraine, irritable bowel syndrome, hyperventilation syndrome, hypertension, autonomic nervous dysfunction, and ulcerative colitis were considered as due to psychosomatic disorder. Students who had past history of hospitalization for at least one of these disorders up to the age of 18 were included in this study.

#### Definitions of intra- or extra-familial adverse childhood experiences

All questions about ACEs pertained to the respondents' first 18 years of life.

### Intra-familial ACE

Emotional abuse, physical abuse, sexual abuse, battered mother, household substance abuse, mental illness in household, parental separation or divorce, and incarcerated household members were defined as adverse childhood experience by Dube et al [8]. In our study, the following six items were deemed as risk factors of poor child-evaluated family function: father's violence toward mother; parental violence toward children; fear of parents; neglect by parents; parental divorce, separation, or death; and the feeling of being unloved by parents during childhood [12]. Based on these factors and the definition of ACEs proposed by Dube et al., we prepared a Japanese version of the questionnaire consisting of 7 items for evaluation of intra-familial ACEs.

#### Witnessing domestic violence

Responses of "often" or " very often" to the question "How often did your father become violent (slap, kick, punch, throw something) toward your mother?" was considered as witnessing domestic violence.

#### Physical violence

Responses of "often" or "very often" to the question "How often did your parents use violence (slap, kick, punch, throw something) against you?" was deemed as experiencing physical violence from parents.

#### Emotional abuse

We used 5 questions to define childhood emotional abuse. (a) Did your parents often neglect you? (b) Did your parents often swear at you? (c)Did your parents often say that you were not necessary for your family? (d) Did your parents regard you as the black sheep of your family? (e) Were you afraid of your parents? All questions were answered with either "yes" or "no", and the emotional score corresponded to the number of "yes" answers. Students who showed emotional scores median + 2SD or more were considered as positive for the experience of emotional abuse.

##### Illness in household

A "yes" response to the question "Were your parents or brothers ill?" defined this ACE.

##### Parental separation or divorce

This experience was defined as a "yes" response to the question "Were your parents ever separated or divorced?"

##### Experience of parental affection

Experience of parental affection was determined from answers to two questions: (a) "Were you loved and treated well by your parents?" and (b) "Do you think that your parents loved you very much?" Responses of "no" to either item determined lack of experience of parental affection.

##### Dysfunctional family

Response of "yes" to the question "Do you think your family was dysfunctional?" was considered as indicative of a dysfunctional family.

#### Extra-familial ACE

Bullying experiences at school reportedly increase psychological stress and subsequently cause depression or physical symptoms, thus decreasing social flexibility of children [13,14]. In addition, it has been reported that negative attitude or recognition of teachers toward students exacerbates their mental discord, probably increasing the frequency of physical complaints [15]. Therefore, we prepared a Japanese version of the questionnaire consisting of the following 5 items for evaluation of extra-familial ACEs.

#### Physical violence from teachers

Responses of "often" or "very often" to the question "How often did teachers use violence (slap, kick, punch, throw something) against you?" indicated ACE.

#### Negative recognition by teachers

A "yes" response to the question "Were you often recognized by teachers negatively?" defined this ACE.

#### Being bullied in elementary or junior high school

This experience was defined as a "yes" response to the question "Were you ever bullied by classmates or upperclassmen in elementary or junior high school?"

#### Sexual violence

A "yes" response to the question "Did you ever experience sexual mischief ?" defined this ACE. Since 95% of sexual mischief originated from unrelated persons, we classified sexual violence as extra-familial ACE.

### Statistical analysis

All analyses were conducted with the SPSS System, Version 11. Pearson's Chi-square was used for categorical data such as prevalence of adverse childhood experience by sex. Adjusted odds ratios for sex and economical state (classified as good, normal, or poor) and 95% confidence intervals (CI) were obtained from logistic regression models that estimated the risk of lifetime history of psychosomatic disorder by each of the 7 intra-familial ACEs and 5 extra-familial ACEs.

The numbers of intra- and extra-familial ACEs were summed for each respondent (intra-familial ACE score range, 0–7; extra-familial ACE score range, 0–5). Because of the small sample size, intra-familial ACE scores of 3, 4, 5, 6, and 7 were combined into 1 category (≥ 3) and extra-familial ACE scores of 3, 4, and 5 were combined into 1 category (≥ 2).

To approximate relative risk (RR) ratios more accurately, we used a simple method to correct the adjusted odds ratios from the logistic regression model [16]. *P *– values below 0.05 were considered to be statistically significant.

## Results

### Frequency of psychosomatic disorder by sex

Two hundred and thirty five (14.8%, males vs. females = 10.1% vs. 19.9%, χ^2 ^= 31.5, p < 0.001) respondents had been hospitalized for psychosomatic disorder at least once up to the age of 18.

### Frequency of each category of intra- and extra-familial ACEs by sex

With regard to intra-familial ACEs, the frequencies of emotional abuse, illness in household, and dysfunctional family were significantly higher in females than in males (χ^2 ^= 17.8, P < 0.001; χ^2 ^= 7.5, P < 0.01; χ^2 ^= 13.5, P < 0.01, respectively), while the frequency of feeling unloved by parents was higher in males than in females (χ^2 ^= 16.5, P < 0.001) (Table 1). With regard to extra-familial ACEs, the frequencies of being bullied at elementary or junior high school or sexual violence were significantly higher in females than in males (χ^2 ^= 31.0, P < 0.001; χ^2 ^= 12.0, P < 0.01; χ^2 ^= 25.1, P < 0.001, respectively), while physical violence from teachers was higher in males than in females (χ^2 ^= 29.8, P < 0.001) (Table 2).

**Table 1:** Prevalence of each category of intra-familial ACE by sex

Intra-familial ACE	Men	Women	Total
	(n = 831)	(n = 761)	(n = 592)
	
	No. (%)	No. (%)	No. (%)
Witnessing domestic violence	85 (10.3)	87 (11.4)	172 (10.8)
Physical violence	45 (5.6)	52 (6.9)	97 (6.2)
Emotional abuse	98 (11.6)	125 (16.0)**	223 (13.7)
Illness in household	91 (11.5)	121 (16.3)*	212 (13.8)
Parental separation or divorce	56 (6.9)	54 (7.3)	110 (7.1)
No experience of affection from parents	72 (8.6)**	37 (4.8)	109 (6.8)
Dysfunctional family	34 (4.0)	47 (6.0)*	81 (5.0)

* P < 0.01, **P < 0.001

**Table 2:** Prevalence of each category of extra-familial ACE by sex

Extra-familial ACE	Men	Women	Total
	(n = 831)	(n = 761)	(n = 1592)
	
	No. (%)	No. (%)	No. (%)
Physical violence from teachers	16 (1.9)	6 (0.8)	22 (1.4)
Negative recognition by teachers	25 (2.9)	35 (4.5)	60 (3.7)
Bullied experience in elementary school	139 (16.4)	216 (27.8)**	355 (21.8)
Bullied in junior high school	77 (9.1)	114 (14.6)*	191 (11.7)
Sexual violence	7 (0.8)	39 (5.0)**	46 (2.9)

### Relative risk of psychosomatic disorder by categories of intra- and extra-familial ACEs

In the 7 intra-familial ACEs, the risk of psychosomatic disorder was increased by emotional abuse (RR = 1.9; 95% CI: 1.4–2.5; p < 0.001) and illness in household (RR = 1.7; 95% CI: 1.3–2.2; p < 0.01) (Table 3). Estimates of the RR for each of the 5 extra-familial ACEs were statistically significant and ranged from 1.5 (95% CI: 1.2–1.9; p < 0.01) for experience of being bullied in elementary school or physical violence by teachers or classmates to 2.4 (95% CI; 1.1–4.0; p < 0.05) (Table 4).

**Table 3:** Prevalence and risk of past history of psychosomatic disorders

Intra-familial ACE Category	Prevalence (%)	Odds ratio (95% CI)	Risk ratio (95% CI)
Witnessing domestic violence			
No	13.1	1.0	1.0
Yes	13.3	0.9 (0.6 – 1.6)	0.9 (0.6 – 1.5)
Physical violence			
No	13.5	1.0	1.0
Yes	15.2	1.1 (0.6 – 2.0)	1.1 (0.6 – 1.8)
Emotional abuse			
No	12.5	1.0	1.0
Yes	25.1	2.2 (1.5 – 3.1)	1.9 (1.4 – 2.5)
Illness in household			
No	13.4	1.0	1.0
Yes	23.2	1.9 (1.3 – 2.7)	1.7 (1.3 – 2.2)
Parental separation or divorce			
No	14.8	1.0	1.0
Yes	14.3	0.9 (0.6 – 1.7)	0.9 (0.6 – 1.5)
No experience of affection from parents			
No	15.6	1.0	1.0
Yes	15.7	1.2 (0.7 – 2.0)	1.2 (0.7 – 1.7)
Dysfunctional family			
No	14.3	1.0	1.0
Yes	24.1	1.7 (0.9 – 2.9)	1.6 (0.9 – 2.3)

**Table 4:** Prevalence and risk of psychosomatic disorders

Extra-familial ACE Category	Prevalence (%)	Odds ratio (95% CI)	Risk ratio (95% CI)
Physical violence from teachers			
No	13.8	1.0	1.0
Yes	28.6	3.0 (1.1 – 7.8)	2.4 (1.1 – 4.0)
Negative recognition by teachers			
No	14.0	1.0	1.0
Yes	27.1	2.3 (1.3 – 4.1)	2.0 (1.3 – 2.9)
Bullied in elementary school			
No	12.9	1.0	1.0
Yes	21.3	1.6 (1.2 – 2.2)	1.5 (1.2 – 1.9)
Bullied in junior high school			
No	13.5	1.0	1.0
Yes	24.1	1.8 (1.3 – 2.7)	1.6 (1.3 – 2.2)
Sexual violence			
No	14.4	1.0	1.0
Yes	26.1	1.6 (0.8 – 3.2)	1.5 (0.8 – 2.4)

### Relationships between the severity of intra- or extra-familial ACEs and relative risk of psychosomatic disorder

Table 5 shows the relationship between the severity of intra- and extra-familial ACEs and the relative risk of psychosomatic disorders. The relative risk of psychosomatic disorder was increased by 2.2 (95% CI: 1.3–3.4; p < 0.01) in students who had 2 or more extra-familial ACEs, even when they did not have any intra-familial ACEs. In addition, the relative risk of psychosomatic disorder was increased by 2.5 in those who had 1 or 2 intra-familial ACEs (95% CI: 1.4–4.0; p < 0.01), and by 3.0 (95% CI: 1.9–4.4; p < 0.01) in those who had 3 or more intra-familial ACEs. In contrast, there was no risk of psychosomatic disorder when students had 2 or more intra-familial ACEs and did not have any extra-familial ACEs. However, the relative risk of psychosomatic disorder was increased by 2.0 (95% CI: 1.1–3.2; p < 0.05) when students had only 1 intra-familial ACE.

**Table 5:** Relationship between relative risk of psychosomatic disorders and intra- and extra-familial ACE

Intra-familial ACE	≧3	1 ~ 2	0
	
Extra-familial ACE	RR (95% CI)	RR (95% CI)	RR (95% CI)
≧2	3.0 (1.9 – 4.4)	2.5 (1.4 – 4.0)	2.2 (1.3 – 3.4)
1	2.0 (1.1 – 3.2)	1.6 (0.9 – 2.6)	0.9 (0.5 – 1.4)
0	1.2 (0.7 – 2.0)	1.1 (0.7 – 1.6)	1.0

## Discussion

Female students had a higher relative risk of developing psychosomatic disorders than males. In senior high school students in Tokyo, the females had more psychosomatic complaints than the males [3]. In the USA, in clinical and epidemiological studies, a consistently higher frequency of depression is noted in women than in men. One factor that might contribute to the high prevalence of depression in women is sex-based violence [17]. The incidence of psychosomatic disorder was higher in females than in males, probably because the frequency of domestic emotional abuse and those who experienced familial disruption were higher in females than in males. Another reason was higher frequencies of female students who had experience of sexual violence or experience of being bullied at elementary or junior high school than males.

We found that emotional abuse and illness in household (intra-familial ACEs) and physical violence, negative recognition by teachers and being bullied in elementary or junior high school (extra-familial ACEs) increased the relative risk of psychosomatic disorder between 1.5 and 2.4-fold in Japanese university students.

Children who are refused by their family members usually show poor levels of self-evaluation and a feeling of powerlessness, and become distrustful of others, resulting in an inability to relate with their surroundings. Patients with functional somatic complaints reported significantly more negative life events, lower self-esteem, more psychophysiologic symptoms and a lower self-evaluation than did patients coming for routine physical examination or health maintenance [18]. It was reported that low parental care was associated with having a lifetime history of depression in employed Japanese adults [19] and poor parental interaction was found to increase somatic complaints in Japanese school children [4]. These findings suggest that emotional abuse during childhood may become a risk factor for developing depression or psychosomatic disorder, as emotionally violated children were not sufficiently cared for by their parents and showed decreased levels of self-esteem.

In schoolchildren, the most important factors associated with psychosomatic complaints were the mother's health and well-being [20]. Parents are usually the most meaningful source of social support in early life, and the perception of parental love and caring have important effects on biological and psychological health and illness throughout life [21]. Caring for a sick member of the family may prevent parents from having spare time or emotional reserves for the healthy family members. Therefore, parents may not be able to show their love to their children sufficiently or provide social and emotional support, probably resulting in increased risk of psychosomatic disorder.

In 1998, the Japanese Ministry of Internal Affairs and Communications reported that the percentage of elementary or junior high school students who had experience of being bullied at school was 31.1% and 27.9%, respectively [22]. In this study, the percentage of students who had experience of being bullied at elementary or junior high school was 22.3% and 12.0%, respectively. Experience of being bullied in elementary or junior high school was low in this study, probably because our study was a retrospective one using university students.

Bullying victims often had physical and psychosomatic health problems [23]. Williams et al. found that 9–10 year olds who reported common health problems such as stomach aches or sleeping problems also reported being victims of bullying two to four times more frequently [24]. According to Hugh-Jones and Smith, one-half of bullied individuals reported as adults long-term effects of being bullied at school, predominantly affecting psychological well being and personal relationships [25]. We found that the children with poor family function tend to be bullied at school as well [26]. If children do not receive sufficient care or emotional support from parents due to poor family function during childhood, they are likely to be bullied at school, as they lack self-confidence and self-assertion. Consequently, the risk of psychosomatic disorder may increase in such children.

Teachers' attitude in school affects children's stress to a great extent. Considerable mental conflict will occur when students, who must be educated by teachers, recognize their teachers negatively. Alexander et al. showed that male students who recognize teachers negatively have more psychosomatic symptoms, thus it is understood that male students repress their desire toward their teachers [15]. This study also made clear that physical violence and negative recognition by teachers increase the risk of psychosomatic disorders.

When students had 2 or more extra-familial ACEs, the risk of psychosomatic disorder was significantly increased (2.2–3.0), regardless of the severity of intra-familial ACEs. However, when students had 3 or more intra-familial ACEs and did not have any extra-familial ACE, the risk of psychosomatic disorder did not increase. These results suggest that the influence of extra-familial ACEs is higher than that of intra-familial ACEs on the onset of psychosomatic disorder.

Since this was a retrospective study using university students, there were some limitations, as follow: it might be hard for the subjects to recall experiences in childhood, and experiences that they did not want to recall might be underestimated or denied. In addition, since we did not investigate the relationship between the development of psychosomatic disorder and the timing of ACEs, more detailed investigations considering chronological factors are needed in the future.

To date, no previous studies have differentiated between intra- and extra-familial ACEs as distinct subcategories of adverse childhood experiences. The results of this study demonstrated that both intra- and extra-familial ACEs are mutually involved in a history of childhood psychosomatic disorder. Therefore, sufficient evaluation and consideration of intra- and extra-familial ACEs are required for prevention and treatment of psychosomatic disorder during childhood and adolescence.

## Abbreviations

ACE = adverse childhood experience; OD = odds ratio; RR = relative risk ratio, CI = confidence intervals, SD = standard deviation
